# The effect of bone morphogenetic protein 2 composite materials combined with cannulated screws in treatment of acute displaced femoral neck fractures

**DOI:** 10.1097/MD.0000000000018976

**Published:** 2020-02-07

**Authors:** Hongwei Gao, Deguo Xing, Zhonghao Liu, Jiachun Zheng, Zhenggang Xiong, Mingzhi Gong, Lan Liu

**Affiliations:** a Department of Trauma and Orthopedics; b Medical Department, The Second Hospital of Shandong University, Shandong Province, Peoples Republic of China.

**Keywords:** bone morphogenetic protein 2, closed reduction, femoral neck fractures, fracture fixation, internal

## Abstract

The risk of avascular necrosis (AVN) and nonunion after treatment of displaced femoral neck fractures is increased in patients aged <60 years. Therefore we established a new protocol for closed reduction and internal fixation (CRIF) using cannulated screws combined with bone morphogenetic protein 2 (BMP-2) composite materials to treat acute femoral neck fractures.

This study enrolled 78 patients with acute femoral neck fractures between April 2014 and September 2016. We treated 46 patients with a mean age of 43.8 years in study group. These patients were treated by CRIF combined with BMP-2 composite materials. In control group, there were 32 patients with a mean age of 42.09 years. The patients were treated by CRIF without BMP-2. The duration between presentation and surgery, operative time, Harris score and complications were recorded.

In study group, 43 patients were followed up with an average of 31.3 months. One patient suffered nonunion and three patients presented AVN. In control group, 28 patients were followed up with an average of 32.3 months, the rate of AVN and fracture nonunion were 25% (7/28) and 21.4% (6/28) respectively, significantly higher than those in study group (*P* < .05).

Acute displaced femoral neck fractures can be treated with CRIF and BMP-2 composite materials in a minimally invasive manner. This technique was reproducible and had fewer complications.

## 1 Introduction

Femoral neck fractures are frequently complicated by avascular necrosis (AVN) of the femoral head and nonunion.^[1]^ The risk of AVN has been reported to be as high as 10% to 43%.^[2,3]^ Nonunion of femoral neck fractures has a reported incidence of 2% to 22% and generally becomes apparent within 1 year.^[4]^ The risk of nonunion is greater with displaced fractures and has been reported to be as high as 30% in some series.^[5]^

The complications may lead to collapse of the femoral head and osteoarthritis. Salvage procedures, such as osteotomy and other treatment options such as vascularized and nonvascularized bone grafts have high failure rates and arthroplasty procedures are not ideal, regarding the patient's young age and higher levels of activity.^[1]^ Lin et al designed a hollow bone graft dynamic hip screw for use in neglected femoral neck fractures.^[6]^ They found it was effective and had fewer complications, but was not less invasive.

RhBMP-2 had been proved to promote fracture healing and had been used in osteonecrosis after femoral neck fracture.^[7]^ Therefore, to pursue an outcome with less invasion and better functional rehabilitation, we established this new protocol to assess:

1) its reproducibility
2) the preliminary clinical and radiographic outcomes
3) the rate of avascular necrosis and nonunion.

Our hypothesis was this new protocol is reproducible and has good clinical and radiographic outcomes with fewer complications.

## 2 Materials and methods

### 2.1 Selection criteria

This was a nonrandomized, retrospective, control study. The inclusion criterion was acute displaced femoral neck fractures and the age of patients was less than 60 years. The exclusion criteria were as follows:

1) patients less than 18 years old;
2) 2)with base of femoral neck fractures or other associated injuries such as femoral shaft fracture;
3) with pathological fractures or serious osteoporosis;
4) with serious medical diseases such as serious heart and cerebrovascular diseases, and could not tolerate operation;
5) we also excluded patients with diabetes mellitus, chronic steroid usage and smoking, because these factors affected bone union.

This study was approved by the ethics committee, and monitored by an independent trial center. All patients participating in this study provided voluntary written informed consent. All clinical investigations have been conducted according to the principles expressed in the Declaration of Helsinki.

### 2.2 Patients

Between April 2014 and September 2016, 284 patients with femoral neck fractures were managed in our hospital. According to the inclusion and exclusion criterion, 78 patients were enrolled in this study and were divided into 2 groups (study group and control group). All patients in study group were consented for use of BMP-2, and there was no donation by the industry. In study group, 46 patients (26 males, 20 females; 18 left, 28 right) were treated by CRIF and BMP-2 composite materials with an average age of 43.8 years. According to Pauwels classification, the fracture pattern included 8 type I, 22 Type II and 16 type III fractures. In control group, 32 patients (17 males, 15 females; 15 left, 17 right) were treated by CRIF without BMP-2 composite materials with an average age of 42.09 years. According to Pauwels classification, the fracture pattern included 5 type I, 13 Type II and 14 type III fractures.

Bone mineral density (BMD) of the femoral neck was measured by dual-energy X ray absorptiometry with use of a DPX-L scanner. Serum sclerostin was assayed on a MesoScale Discovery, utilizing a proprietary combination of electrochemiluminescence detection and patterned arrays. Body Mass Index (BMI) was calculated according to height and weight of patients.

### 2.3 Surgical technique

**Figure 1:**
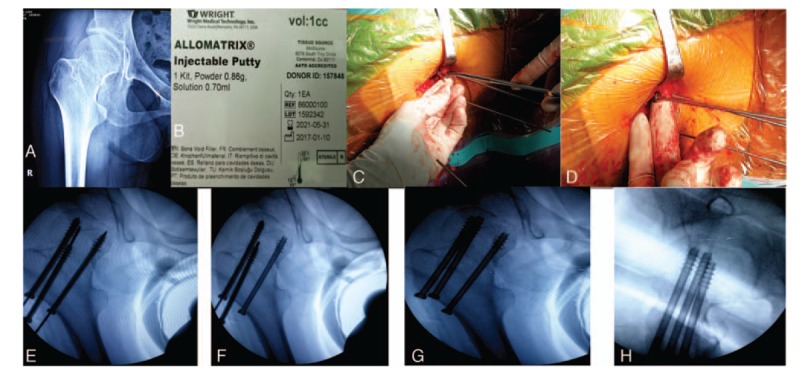
Protocol using bone morphogenetic protein 2 (BMP-2) composite materials passed through cannulated screws into the fracture site. (A) Preoperative radiograph. (B) BMP-2 composite materials (C,D,E,F) were placed in the last cannulated screw at the fracture site, the guidewire was removed, and BMP-2 composite materials were pushed into the fracture site. (G,H) Postoperative radiograph.

The surgical techniques of the study group were briefly described as follows. Under general or epidural anesthesia, patients were placed on a fracture table in supine position. Typically, a displaced femoral neck fracture can be treated with CRIF.^[8]^ Definitive fixation was performed with three 7.3-mm cannulated cancellous screws (Weigao Orthopedic Materials Co., Weihai, China) along the axis of the femoral neck. These were placed in a triangular pattern, with 2 superior screws and 1 inferior screw parallel to each other. We first placed the inferior screw and 1 superior screw on the appropriate site, and the third screw was positioned at the fracture site under fluoroscopic control. BMP-2 composite materials (Allomatrix, Wright Medical Technology, Inc. USA) were made into strips and inserted into the last cannulated screw. We pushed 1 cc of BMP-2 composite materials into the fracture site with a 3.5-mm Kirschner wire through the screw hole, and then placed the last cannulated screw at the correct site (Fig. 1).

In control group, the surgical technique is similar to that in study group, except that BMP-2 composite materials is not required. The duration between presentation and surgery, operative time and blood loss were recorded and analyzed.

### 2.4 Postoperative treatment and follow-up assessment

**Figure 2:**
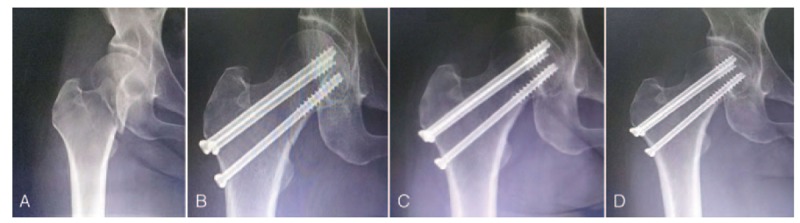
Fluoroscopic anteroposterior view of the displaced femoral neck fracture fixed with parallel cannulated screws, taken at different times. (A) Preoperative X-ray, (B) postoperative X-ray on the day of surgery, (C) postoperative X-ray at 6 months, (D) postoperative X-ray at 15 months.

Anteroposterior and lateral radiographs of the injured femoral neck were taken, and the fracture reduction was evaluated using the method proposed by Garden.^[9]^ Clinical and radiological evaluation was performed at 3 to 12 months after surgery. The Harris hip score (HHS) was determined. Union was assessed if the fracture gap disappeared and the patient could walk with full weight-bearing without pain (Fig. 2). Avascular necrosis was assessed according to the criteria of Ficat.^[10]^

### 2.5 Statistical analysis

First, we made power analysis with the use of G∗Power version 3.0.10 for two group independent sample t-test. The power analysis showed the expected sample size of group was 45, and we inputed the actual sample size and calculated, the power was 0.73. Then the data were analyzed with the use of SPSS 16.0 for Windows (SPSS Inc., Chicago, IL). Categorical variables were recorded as the number and percentile with frequency tables, which were analyzed using the Chi-square test. The Continuous variables with normal distributions were expressed as the mean ± standard deviation (SD), and analyzed using the 2 sample *t* test. Continuous variables with non-normal distributions were recorded as the median and interquartile values, and analyzed using the Mann-Whitney *U* test. A *P* < .05 was considered statistically significant.

## 3 Results

A total of 78 patients were enrolled in this study, there were 46 patients in study group and 32 patients in control group. Table 1 describes the patient demographics. Patient age and sex, BMD, BMI, Serum sclerotin level, fracture side, injury mechanism and fracture classification were compared, and no significant differences were found between groups (all *P* > .05).

**Table 1:**
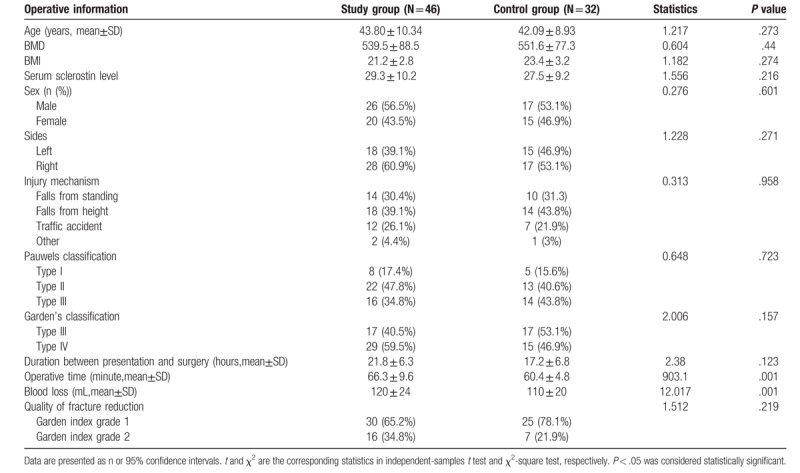
Operative information of patients in study and control groups.

The median duration between presentation and surgery was 21.8 hours in study group and 17.2 hours in control group (*P* > .05). The average operative time and blood loss were 66.3 minutes and 120 mL in study group, and 60.4 minutes and 110 mL in control group, respectively (both *P* < .05).

All fractures were reduced in a closed fashion and fixed with three parallel cannulated screws, except BMP-2 composite materials were used in study group. Femoral neck fracture reductions were assessed on postoperative radiographs. In study group, 30 reductions were assessed as Garden index grade I and 16 as grade 2. In control group, 25 reductions were assessed as Garden index grade I and 7 as grade 2. There was no significant difference between groups in the quality of fracture reduction (*P* > .05).

In study group, 43 patients were available for the final evaluation, and the average follow-up time was 31.3 months (Table 2). Three patients experienced AVN of the femoral head, and one presented nonunion. The mean Harris score was 89.1. Twenty-eight patients were followed up for an average of 32.3 months in control group. Patient age, sex and follow-up period were comparable between groups (all *P* > .05). However, the mean Harris score in control group was 85.6, significantly lower than that in study group (*P* < .05). The rates of AVN of the femoral head and fracture nonunion in control group were 25% (7/28) and 21.4% (6/28), respectively, significantly higher than those in study group (both *P* < .05).

**Table 2:**
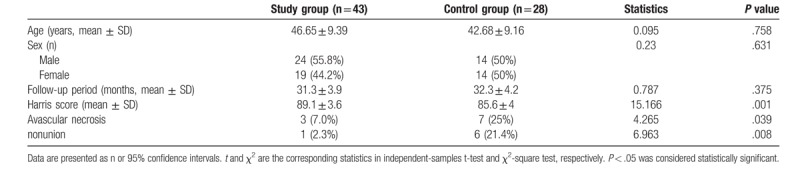
Clinical outcomes of the patients in both groups.

When AVN and nonunion happened, we conducted revision surgery. Four nonunion cases in control group were revised to total hip arthroplasty, and 2 nonunion cases in control group and 1 case in study group were revised to hemi-arthroplasty. For avascular necrosis, 6 in control group and one in study group were revised to arthroplasty, three other cases were not revised.

## 4 Discussion

Femoral intracapsular neck fractures remain challenging for the surgeon, particularly in the young population.^[11–13]^

This study investigated the outcomes of acute displaced femoral neck fractures treated with CRIF and augmented with BMP-2 composite materials. Only one patient suffered nonunion and received arthroplasty, and three patients presented avascular necrosis in study group, significantly lower than those in control group (*P* < .05). fewer complications were achieved in study group. Our results were similar to those of Yu.^[14]^ However, Yu used vascularized iliac bone graft. This technique was not less invasive and required extensive microsurgery, making it difficult to generalize the results. Overall, our hypothesis was confirmed.

The mean Harris hip score was 89.1 in study group, significantly higher than those in control group (*P* < .05). But the HHS was 85.6 in control group, the difference between the 2 groups was 3.5, it is lower than Minimal clinically important difference (MICD), so there was no significant clinically difference on function.^[15]^ And the functional recovery in study group should be investigated in future studies.

Displaced femoral neck fractures have a significant rate of poor outcomes due to a high incidence of complications, among which nonunion and AVN of the femoral head are the 2 most commonly encountered and are intractable.^[1,16]^ Despite advances in treatment, Nonunion rates are reported to range between 4% and 15% after nondisplaced fractures and even 4% to 40% after displaced fractures.^[17–19]^ Park^[20]^ analyzed 1133 femoral neck fractures retrospectively and reported a nonunion incidence of 30.1% for displaced fractures vs. a nonunion rate of 8.5% for nondisplaced fractures.

The risk of avascular necrosis is another main concern in femoral neck fracture treatment with internal fixation. According to Japanese clinical guidelines, avascular necrosis occurs in 4% to 21% of nondisplaced fractures and 46% to 57% of displaced fractures.^[21]^ In a meta-analysis by Xu et al, the avascular necrosis rate for nondisplaced femoral neck fractures in the conservatively treated group was 10.3%, and in the surgically treated group, 7.7%.^[22]^ Therefore, we selected patients with displaced femoral neck fractures as subjects in this study to observe the effect of CRIF with BMP-2 composite materials. Nondisplaced fractures were excluded from this study to reduce the risk of unnecessary grafting procedures.

Various treatment algorithms, including muscle-pedicle-bone grafting and vascularized or free bone grafting, have been developed to reduce the complication rate of femoral neck fractures after internal fixation. Bone grafting with internal fixation had emerged as a reliable method with good long-term functional outcomes.^[23]^ Dewei et al^[24]^ treated displaced subcapital and transcervical fractures of the femoral neck using vascularized iliac bone grafts and internal fixation in an open manner. However, the technique of vascularized pedicle grafting is highly technical and requires microsurgical facilities and experience. Li et al^[25]^ treated displaced femoral neck fractures with free iliac bone grafting and cannulated screws and also achieved a satisfactory result. To reduce the iatrogenic damage of an open operation and the requirement for special screws, we treated acute displaced femoral neck fractures using BMP-2 composite materials in a minimally invasive fashion.

A major advantage is that this technique used BMP-2 composite materials based on three cannulated screws and does not need another operation for bone grafting. We often fixed the inferior and superior cannulated screws, and then passed BMP-2 composite materials to the fracture site through the last cannulated screw. When displaced femoral neck fractures accompanied comminution of the posteromedial cortex of the femoral neck, which is an important risk factor for nonunion due to the loss of the buttressing effect against lateral rotation and insecure fixation,^[26]^ we first fixed the 2 superior screws and passed BMP-2 composite materials into the comminuted fracture site through the inferior cannulated screw for healing of the posteromedial cortex of the femoral neck.

There are 3 major limitations of our study. First, this was a nonrandomized retrospective study and the sample size was small, which may increase the risk of type II error. A randomized controlled trial with a larger sample size and longer follow-up period should be performed. Second, the patients older than 65 years were excluded in this study. For the elderly, it remains a matter of debate whether displaced femoral neck fractures should be treated with internal fixation or arthroplasty.^[27]^ Third, we did not analyze the cost-effectiveness ratio of the surgical methods, and we will evaluated it on the basis of this manuscript.

## 5 Conclusions

We treated acute displaced femoral neck fractures using CRIF with BMP-2 composite materials. BMP with cannulated screws can provide fewer complications including avascular necrosis and nonunion. The change of HHS needs large sample study to assess. So this technique may be an effective and less invasive alternative for displaced femoral neck fractures.

## Acknowledgments

The authors would like to thank Yuan Yu for language help and Jie Yang for Statistical analysis.

## Author contributions

**Conceptualization:** Zhonghao Liu, Mingzhi Gong, Lan Liu.

**Data curation:** Jiachun Zheng.

**Formal analysis:** Jiachun Zheng.

**Funding acquisition:** Hongwei Gao.

**Investigation:** Deguo Xing, Lan Liu.

**Methodology:** Deguo Xing, Zhenggang Xiong, Lan Liu.

**Project administration:** Hongwei Gao, Zhenggang Xiong, Mingzhi Gong.

**Resources:** Zhonghao Liu, Jiachun Zheng.

**Validation:** Zhenggang Xiong.

**Visualization:** Deguo Xing.

**Writing – original draft:** Hongwei Gao.

**Writing – review & editing:** Hongwei Gao, Zhonghao Liu, Mingzhi Gong, Lan Liu.
